# Successful Management of Pheochromocytoma Detected in Pregnancy by Interval Adrenalectomy in a VHL Patient

**DOI:** 10.1155/2018/9014585

**Published:** 2018-07-19

**Authors:** V. T. S. Kaluarachchi, Uditha Bulugahapitiya, Maulee Arambewela, Sonali Gunathilake

**Affiliations:** ^1^Colombo South Teaching Hospital, Kalubowila, Sri Lanka; ^2^National Hospital of Sri Lanka, Colombo, Sri Lanka

## Abstract

A 34-year-old mother with diabetes mellitus for 6 years presented in the late second trimester of her third pregnancy with new onset hypertension and characteristic hyperadrenergic spells. Clinical examination was unremarkable except a blood pressure of 170/110 mmhg. She had an elevated 24 hour urinary normetanephrine level with ultrasonic evidence of a hyperechoic hypervascular well-defined right supra renal mass of 6 x 5 cm in size which was very suggestive of a pheochromocytoma. Her management decisions were made by a multidisciplinary team which decided to deliver the baby by lower segment cesarean section (LSCS) as the pregnancy was advanced and to proceed with interval adrenalectomy after contrast enhanced computer tomography (CECT) of the abdomen with adrenal protocol. As a result a healthy baby was delivered by an uncomplicated elective LSCS at 36 weeks of POA. CECT abdomen with adrenal protocol confirmed a right-sided pheochromocytoma without any evidence of metastasis. Uncomplicated laparoscopic right adrenalectomy led to a clinical and biochemical recovery of the patient while histology confirmed the pheochromocytoma without any evidence of invasion. Subsequent follow up revealed cerebellar hemangioblastomas and retinal angioma in the right eye which led to a clinical diagnosis of Von Hippel Lindau disease (VHL). Even though clinical criteria for Von Hippel Lindau disease were fulfilled, her VHL genetic test was negative. At present she and her family are under surveillance of the endocrine team.

## 1. Introduction

Hypertension is a common complication during pregnancy which causes significant morbidity and mortality to the mother as well as to the foetus. Some common aetiologies of hypertension during pregnancy are pre-eclampsia, gestational hypertension, and essential hypertension. Pheochromocytoma is a rare aetiological diagnosis of hypertension during pregnancy occurring in 0.007% of all pregnancies [1]. In fact this would cause potential life-threatening cardiovascular complications to the mother and foetus while increasing maternal and foetal mortality by 8% and 17%, respectively. However, early diagnosis and timely, appropriate management by multidisciplinary approach reduce these maternal and fetal complications.

Diagnosis of pheochromocytoma during pregnancy is challenging as some clinical features are common to normal pregnant state (e.g.: tachycardia, increased sweating). In addition to these common features one discriminating feature in pregnant patient is paradoxical supine hypertension with normal erect and sitting blood pressure, due to the pressure of the gravid uterus on the tumor during supine position.

In addition, Von Hippel Lindau disease is a rare autosomal-dominant genetic disorder with variable penetrance which is characterized by hemangioblastomas in brain, spinal cord and retina, pheochromocytomas, and multiple renal and pancreatic cysts with an increased risk of malignant transformation. Diagnosis of this disease can be done clinically as well as with the detection of the VHL gene.

## 2. Case

A 34-year-old mother of two children presented in 29^th^ week of her third pregnancy with hypertension. She is a diagnosed patient with diabetes mellitus for 6 years. However her previous two pregnancies, which were two and seven years back, were not complicated with hypertension or diabetes mellitus. She was not detected to have hypertension until the early third trimester of this pregnancy despite frequent clinic visits where blood pressure measurement was a routine practice. She described episodic palpitation, headache, and sweating suggestive of hyperadrenergic spells associated with episodes of high blood pressure. At the same time, her blood pressure and blood sugar levels were fluctuating and difficult to control with her usual medications. On examination, she was an averagely built lady with no features suggestive of syndromic associations (such as mucosal neuromas, café au lait spots, axillary or inguinal freckling, and iris hamartomas). Cardiovascular system examination was unremarkable except for a blood pressure of 170/110 mmHg. Ophthalmoscopic evaluation did not revealed retinal angiomas. Her 24 hour urinary vanillin mandelic acid level was 22.2mg/24hrs (1-13.6 mg/24hrs), 24 hour urinary metanephrine was 188.2*µ* g/24hrs (<350 *µ* g/24hrs), and 24 hour urinary normetanephrine was 653*µ* g/24hrs (<600 *µ* g/24hrs).

Ultrasound scan of the abdomen done at 29 weeks of POA by consultant radiologist showed a hyperechoic hypervascular well-defined right supra renal mass which is in contact with but not invading the right kidney. The ultrasonic appearance was suggestive of a pheochromocytoma and there were no lesions suggestive of metastasis elsewhere in the abdomen. Patients' management decisions were taken by a multidisciplinary team meeting held with the participation of the surgeon, radiologist, obstetrician, neonatologist, anesthetist, and the endocrinologist where they decided to proceed with an interval adrenalectomy to avoid the detrimental complications of handling hypervascular tumor which was closer to the liver at the time of the caesarean delivery. Her blood pressure control was achieved by an alfa adrenergic blocker (initially prazosin and later phenoxybenzamine) followed by a beta adrenergic blocker which was started after adequate alfa blockade. Blood sugar control was optimized with basal bolus regimen of insulin. Her delivery was done at the 36 weeks of gestation by an elective LSCS under close supervision for adrenergic crisis. Peripartum period was uneventful and there was no adrenal crisis. She gave birth to a healthy baby of a birth weight of 3.6kg. CECT abdomen with adrenal protocol was planned after one month postpartum. She was discharged on the 12^th^ postpartum day after close maternal and neonatal surveillance. The patient and her family were well educated regarding the disease and the need for close monitoring of blood pressure. Her blood pressure was monitored weekly at the hospital. She was well compliant on twice a day premixed insulin and self-monitoring of blood sugar.

**Figure 1:**
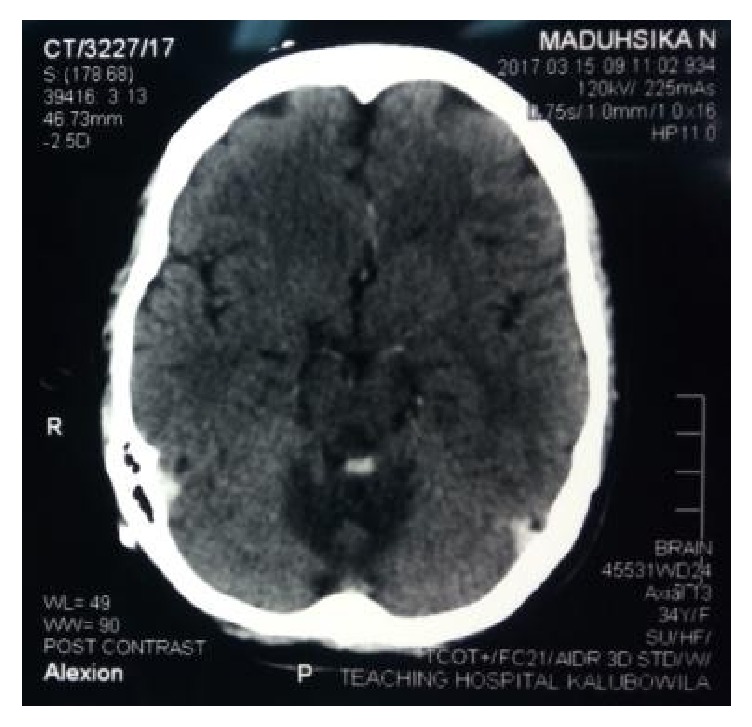
Noncontrast CT brain showed early hydrocephalus with a suspicion of a posterior fossa space occupying lesion.

20 days after the delivery she presented with a sudden onset severe headache with vomiting. Blood pressure was normal and fundi did not revealed papilloedema. Noncontrast CT brain (Figure 1) showed early hydrocephalus with a suspicion of posterior fossa space occupying lesion.

**Figure 2:**
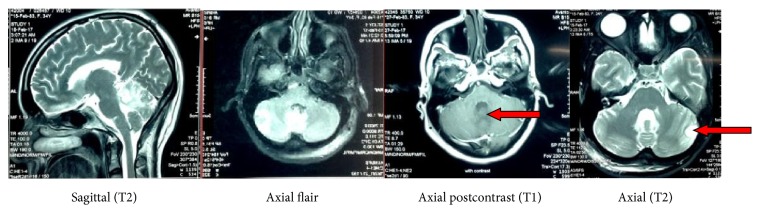
Multiple cystic lesions in cerebellum with enhancing neural nodules and mild perilesional oedema suggestive of hemangioblastomas (central and peripheral lesions are indicated by red arrows).

She was transferred to neurosurgical unit and contrast MRI brain (Figure 2) showed two multiloculated cystic lesions one in relation to the fourth ventricle and the vermis and the other lesion in the periphery of the left cerebellar hemisphere which were suspicious of cerebellar hemangioblastomas. She underwent urgent ventriculoperitoneal shunting as an emergency procedure to relieve intracranial pressure while awaiting definitive management.

**Figure 3:**
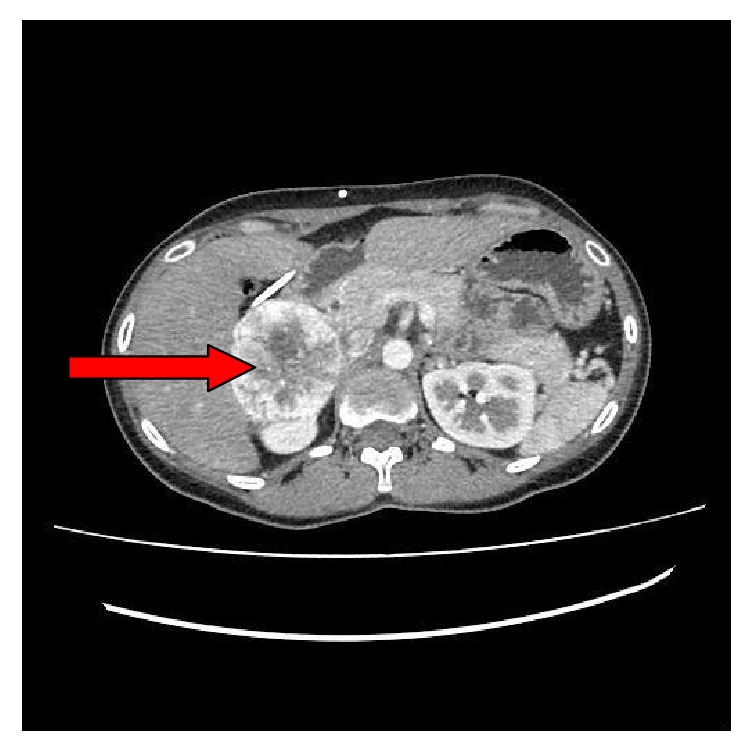
Well-defined avidly enhancing (190 HU) mass lesion 66 x 59 mm in the region of R/suprarenal gland. There is a centrally nonenhancing component suggestive of necrosis (red arrow). No evidence of metastasis.

CECT abdomen with adrenal protocol (Figure 3) showed a right-sided adrenal tumor of 66×59mm size which is suggestive of a pheochromocytoma without any evidence of metastatic deposits in the abdomen.

She was started on Phenoxybenzamine 14 days before surgery for the optimum control of blood pressure and adequate alfa blockade to prevent adrenergic crisis.

Laparoscopic right adrenalectomy was performed without any major intraoperative or postoperative complications. This caused the reversal of all the clinical features of hyperadrenalism. Her antihypertensives were stopped after the surgery and her diabetic control improved. Her postoperative investigations are shown in Table 1.

Histology confirmed the diagnosis of pheochromocytoma.

She was evaluated for the syndromic associations particularly for von Hippel Lindau disease because of the presence of cerebellar lesions suggestive of hemangioblastomas.

**Figure 4:**
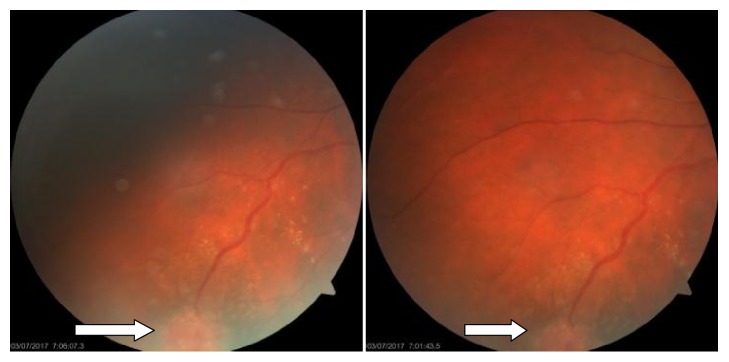
Retinal angioma (white arrow) with a dilated feeding vessel.

Ophthalmology assessment revealed a lesion suggestive of a retinal hemangioblastoma (retinal angioma) on the right eye (Figure 4). It was treated with laser therapy and currently under ophthalmology followup.

VHL genetic testing was found to be negative. Her two brothers and three children are currently under evaluation.

She is under close surveillance at neurosurgical clinic with regular MRI. The resection of deeply seated cerebellar haemangioma without any symptoms or enlargement over time may have adverse consequences neurosurgical team decided to closely follow up the patient and go for surgery when indicated. At 6 months, MRI did not show significant increase in tumor size and she is asymptomatic.

Following the surgery we were able to stop all the antihypertensive medications and her blood glucose is under control with only one oral antidiabetic medication (metformin).

## 3. Discussion

Pheochromocytomas and paragangliomas are rare catecholamine-secreting tumors arising from the chromaffin cells of the adrenal medulla or sympathetic ganglia, respectively. Excess release of catecholamines from the tumor (epinephrine, norepinephrine, and to a lesser extent dopamine) causes excessive stimulation of the sympathetic nervous system which causes elevated blood pressure. Many adrenal tumors produce both norepinephrine and epinephrine but most (90%) extra-adrenal tumors produce predominantly norepinephrine [2].

Epinephrine-secreting pheochromocytomas produce episodic symptoms and signs, such as palpitations, lightheadedness or syncope, anxiety, and hyperglycemia; norepinephrine-producing tumors are more often associated with continuous symptoms and signs.

The diagnosis of catecholamine producing tumors in pregnant patients is often missed because the symptoms and signs can mimic other forms of hypertension such as new onset hypertensive syndromes in pregnancy, gestational hypertension, and preeclampsia. The appearance of hypertension can be insidious, and the patient may even appear asymptomatic until delivery. Therefore, careful history including detailed family history to detect any hereditary syndromes and clinical examination is mandatory in a patient presenting with hypertension during pregnancy.

Few differences between pregnancy induced hypertension and pheochromocytoma are shown in Table 2. [3]

The classic triad of symptoms in patients with a pheochromocytoma are

1. episodic headache,
2. sweating,
3. tachycardia.

Most patients with pheochromocytoma may not have the classic triad of symptoms. The prevalence of these symptoms in a pregnant patient is probably slightly lower than in nonpregnant patients [4]. About 5 to 15 percent of patients present with normal blood pressure. As in our patient, enlarging uterus, changes of intra-abdominal pressure during fetal movements and compression of the growing tumor by pregnancy may lead to the release of catecholamines and the occurrence of spells. In addition to hypertension, altered renal function and proteinuria are common in pheochromocytoma which may be caused by catecholamine-mediated renovascular abnormalities [5]. Hyperglycaemia, which is caused by adrenalin, can be a presenting feature and can be recognized as gestational diabetes or chronic diabetes, which was evident in our case.

As in nonpregnant women, the diagnosis is usually based upon the results of 24-hour urinary fractionated metanephrines (metanephrine and normetanephrine) and catecholamines (dopamine, norepinephrine, and epinephrine) and plasma fractionated metanephrines [6]. The 24-hour urinary vanillyl mandelic acid (VMA) excretion is considered to be less sensitive and specific compared with fractionated 24-hour urinary fractionated metanephrines. Interfering medications should be stopped at least two weeks before the test (tricyclic antidepressants, levodopa, etc.).

Imaging test of choice in the pregnant woman is the MRI without gadolinium. Stimulation tests and 123-I-MIBG scintigraphy are contraindicated for pregnant women.

When the diagnosis of pheochromocytoma was made during the antenatal period better fetal and maternal outcome is expected than when it was made during labor or the immediate postpartum period where it would be associated with higher fetal and maternal mortality.

Medical treatment with alfa- and beta-adrenergic blockers during pregnancy is similar to nonpregnant adults (pregnancy category C). Alpha-adrenergic blockade followed by beta-adrenergic blockade is recommended to prevent alfa receptor overstimulation by catecholamine action on alfa receptors. Alfa blockers are recommended as the first choice of preoperative treatment to prevent cardiovascular complications in patients with pheochromocytoma. There is no randomized control trial evidence regarding the comparable effectiveness between nonselective alfa adrenergic receptor blockers (phenoxybenzamine) and alfa-1 selective adrenergic receptor blockers (prazosin, doxazosin) for preoperative preparation[6, 7]

Timing of the surgery for pheochromocytoma is controversial during pregnancy. If the foetus is previable (<24 weeks of gestation) surgical removal of the tumor during pregnancy and if the pregnancy is advanced medical management until foetal lung maturity is documented in the literature. This is based in the concept of the fetal distress and hypotension documented following prolonged treatment of phenoxybenzamine [8].

Mode of delivery which is preferred in a patient with a pheochromocytoma is the cesarean section as it carries a lesser risk of maternal death [9]. Combined cesarean section and tumor resection nearer to term is the procedure of choice in the patients who are medically managed during pregnancy; however the postpartum removal of the tumor as in our patient is also possible according to the literature [2, 9]. Our patient had stable blood pressure during postpartum period with adequate alfa blockade with prazosin and beta blockade with propranolol. Preferred surgical option is minimally invasive adrenalectomy when it is planned postpartum if the tumor is small (<6cm) and there are no features of invasion. Our patient underwent laparoscopic right adrenalectomy at postpartum sixth week without any intraoperative or postoperative complications.

Measuring plasma or urine levels of metanephrines at 2-4 weeks postsurgery is recommended to determine the successful tumor removal [6]. Also the lifelong annual biochemical testing is recommended to assess for the recurrence or metastatic disease. Our patient had normal normetanephrine level at 4 weeks after surgery indicating successful tumor removal.

All patients presenting with pheochromocytoma should undergo genetic testing according to the 2014 Endocrine Society Clinical Practice Guidelines [6]. This is because at least one-third of the patients diagnosed with pheochromocytoma have germ line mutations [10]. Because of the financial concerns the genetic testing should be done according to a clinical feature driven algorithm. Clinical features that indicate a high likelihood of a hereditary cause include a positive family history, syndromic features and multifocal, bilateral, or metastatic disease. Our patient had all the clinical criteria for the diagnosis of VHL (Von Hippel Lindau disease).

Von Hippel-Lindau (VHL) disease is an autosomal dominant syndrome associated with a variety of benign and malignant tumors. Prevalence of VHL gene abnormality is approximately 1 in 36,000 individuals [11]. The mean age of the initial presentation is approximately 26 years; however initial manifestations of disease can occur in childhood, adolescence, or adulthood [11]. VHL is associated with the following tumors [12]:

- Hemangioblastomas of the brain, spinal cord, and retina
- Renal cysts and clear cell renal cell carcinoma
- Pheochromocytoma
- Pancreatic cysts and neuroendocrine tumors
- Endolymphatic sac tumors
- Epididymal and broad ligament cysts

Among these the retinal angiomas may be the earliest presentation in adults while renal cell carcinoma is the commonest cause of death.

Criteria for the diagnosis of VHL are as follows [12]:

- An individual with no known family history of VHL syndrome (simplex case) presenting with two or more characteristic lesions:
  1. Two or more hemangioblastomas of the retina, spine, or brain or a single hemangioblastoma in association with a visceral manifestation (e.g., multiple kidney or pancreatic cysts)
  2. Renal cell carcinoma
  3. Adrenal or extra-adrenal pheochromocytomas
  4. Endolymphatic sac tumors, papillary cystadenomas of the epididymis or broad ligament, or neuroendocrine tumors of the pancreas-less common
- An individual with a positive family history of VHL syndrome in whom one or more of the following manifestations is present:
  1. Retinal angioma
  2. Spinal or cerebellar hemangioblastoma
  3. Adrenal or extra-adrenal pheochromocytoma
  4. Renal cell carcinoma
  5. Multiple renal and pancreatic cysts

VHL is the result of a germline mutation of the VHL gene which is located on the short arm of chromosome 3 [13]. This gene product, pVHL, functions as a tumor suppressor protein.

Genetics and pattern of inheritance [12] are

1. autosomal dominant with 100% penetrance–80%
2. de novo mutation–20%
3. somatic mosaicism—incidence not known

Genetic testing for VHL is usually done on peripheral blood lymphocytes which can be negative if the patient expresses mosaicism for the VHL gene. There are several methods to detect VHL gene and our patient had negative genetic testing (sequence analysis method) even though she has characteristic clinical features.

Possibilities when a sequence variant is not detected during sequence analysis are as follows:

1. Patient does not have a pathogenic variant in the tested gene (e.g., a sequence variant exists in another gene at another locus).
2. Patient has a sequence variant that cannot be detected by sequence analysis (e.g., a large deletion).
3. Patient has a sequence variant in a region of the gene (e.g., an intron or regulatory region).

Above aspects are not covered by the sequence analysis method [12].

Usually the germ line mutations of VHL gene are inherited. If it occurs during embryonic life after fertilization it will lead to somatic mosaicism. In this situation, some cells will be normal while other cells carry the mutation giving rise to diagnostic difficulties [14]. In this situation an individual may present with classic VHL features but the mutation may not be detectable in the peripheral blood as the hematologic stem cells do not carry the mutation. The manifestations depend on the timing of the mutation event during embryonic life. If the mutation event occurred early in the embryonic life more tissue types are likely to get affected.

Another cause for negative genetic test is the presence of a novel mutation which is not detected by the available genetic test.

### 3.1. VHL and Pregnancy

VHL related lesions may demonstrate accelerated growth in some women during pregnancy (patients who have a prior history of pheochromocytomas, CNS lesions, or retinal lesions) [15]. Eyes should be checked regularly throughout the pregnancy, and a noncontrast MRI may be considered in the fourth month to follow up on CNS lesions. Plasma metanephrines should be done at early, mid, and late pregnancy to exclude pheochromocytoma.

### 3.2. Genetic Counselling

If the patient is genetic test positive they should be subjected to appropriate genetic counselling as the disease is autosomally dominant. Affected individuals have a 50 percent probability of transmitting the VHL mutation to each offspring. If the patient has somatic mosaicism the risk to offspring depends on whether or not the germ tissue carries the mutation. As the somatic mosaicism cannot be clinically diagnosed those patients should be informed that the risk of the offspring to get VHL may be as high as 50% and the offspring will have severe disease as 100% of their cells carry the mutation.

### 3.3. Surveillance Protocol [16]

Surveillance is the only way to prevent severe VHL complications as there is no cure found for VHL. Improvement of the understanding of the natural history of serious manifestation of the VHL syndrome has led to substantial reduction of morbidity and mortality. Surveillance has focused primarily on hemangioblastomas, retinal angiomas, RCCs, and pheochromocytomas.

This should be carried out in the patients who are already diagnosed and there family members at risk.

## Figures and Tables

**Table 1:** Postoperative investigations.

Test	Pre OP	Post OP	Reference ranges
24 hour urinary VMA	22.2mg/24hrs	8.6mg/24hrs	1-13.6 mg/24hrs

24 hour urinary metanephrines	188.2*μ* g/24hrs	Not done	<350 *μ* g/24hrs

24 hour urinary normetanephrines	653*μ* g/24hrs	398 *μ* g/24hrs	<600 *μ* g/24hrs

**Table 2:** 

**Feature**	**Pregnancy induced hypertension**	**Pheochromocytoma**
Hypertension	Not paroxysmal	Paroxysmal

Onset of hypertension	Usually after 20 weeks of gestation	Any time during pregnancy

Other symptoms	ankle edema, proteinuria and an elevated plasma uric acid are common	ankle edema, proteinuria and an elevated plasma uric acid are not common

Orthostatic hypotension	uncommon	Common

Syndromic features signs of NF1- café au lait spots, skin freckling or cutaneous fibromas	uncommon	Can be seen

## References

[1] Harrington J. L., Farley D. R., Van Heerden J. A., Ramin K. D. (1999). Adrenal tumors and pregnancy. *World Journal of Surgery*.

[2] Bravo E. L., Tagle R. (2003). Pheochromocytoma: State-of-the-art and future prospects. *Endocrine Reviews*.

[3] Lenders J. W. M. (2012). Pheochromocytoma and pregnancy: a deceptive connection. *European Journal of Endocrinology*.

[4] Ahlawat S. K., Jain S., Kumari S., Varma S., Sharma B. K. (1999). Pheochromocytoma associated with pregnancy: case report and review of the literature. *Obstetrical & Gynecological Survey*.

[5] Huddle K. R. L., Nagar A. (1999). Phaeochromocytoma in pregnancy. *Australian and New Zealand Journal of Obstetrics and Gynaecology*.

[6] Lenders J. W. M., Duh Q.-Y., Eisenhofer G. (2014). Pheochromocytoma and paraganglioma : an endocrine society clinical practice guideline. *The Journal of Clinical Endocrinology & Metabolism*.

[7] Oliva R., Angelos P., Kaplan E., Bakris G. (2010). Pheochromocytoma in pregnancy: a case series and review. *Hypertension*.

[8] Santeiro M. L. (1996). Phenoxybenzamine placental transfer during the third trimester. *Annals of Pharmacotherapy*.

[9] Takahashi K., Sai Y., Nosaka S. (1998). Anaesthetic management for Caesarean section combined with removal of phaeochromocytoma. *European Journal of Anaesthesiology*.

[10] Buffet A., Venisse A., Nau V. (2012). A decade (2001-2010) of genetic testing for pheochromocytoma and paraganglioma. *Hormone and Metabolic Research*.

[11] Maher E. R., Yates J. R. W., Harries R. (1990). Clinical features and natural history of von hippel-lindau disease. *QJM: An International Journal of Medicine*.

[12] Frantzen C., Klasson T. D., Links T. P., Giles R. H., Adam M. P., Ardinger H. H., Pagon R. A. (2000). Von Hippel-Lindau Syndrome. *GeneReviews® [Internet]*.

[13] Latif F., Tory K., Gnarra J. (1993). Identification of the von Hippel-Lindau disease tumor suppressor gene. *Science*.

[14] Sgambati M. T., Stolle C., Choyke P. L. (2000). Mosaicism in von Hippel-Lindau disease: Lessons from kindreds with germline mutations identified in offspring with mosaic parents. *American Journal of Human Genetics*.

[15] Frantzen C., Kruizinga R. C., Van Asselt S. J. (2012). Pregnancy-related hemangioblastoma progression and complications in von Hippel-Lindau disease. *Neurology*.

[16] VHLA Suggested Active Surveillance Guidelines. http://www.vhl.org.

